# Monte-Carlo simulation for calculating phakic supplementary lenses based on a thick and thin lens model using anterior segment OCT data

**DOI:** 10.1007/s00417-023-06331-7

**Published:** 2023-12-27

**Authors:** Achim Langenbucher, Alan Cayless, Kitti Kormanyos, Jascha Wendelstein, Peter Hoffmann, Nóra Szentmáry

**Affiliations:** 1 Department of Experimental Ophthalmology, Saarland University, Kirrberger Str 100 Bldg. 2266424, Homburg/Saar, Germany; 2 School of Physical Sciences, The Open University, Milton Keynes, UK; 3 Department of Ophthalmology, Semmelweis-University, Budapest, Hungary; 4 Department of Ophthalmology and Optometry, Johannes-Kepler University, Linz, Austria; 5 Augen- und Laserklinik Castrop-Rauxel, Castrop-Rauxel, Germany; 6 Dr. Rolf M. Schwiete Center for Limbal Stem Cell and Aniridia Research, Saarland University, Homburg/Saar, Germany

**Keywords:** Phakic lens, Vergence calculation, Thick lens model, Refraction correction, Ocular magnification, Anterior segment tomography

## Abstract

**Background:**

Phakic lenses (PIOLs, the most common and only disclosed type being the implantable collamer lens, ICL) are used in patients with large or excessive ametropia in cases where laser refractive surgery is contraindicated. The purpose of this study was to present a strategy based on anterior segment OCT data for calculating the refraction correction (REF) and the change in lateral magnification (Δ*M*) with ICL implantation.

**Methods:**

Based on a dataset (*N* = 3659) containing Casia 2 measurements, we developed a vergence-based calculation scheme to derive the REF and gain or loss in Δ*M* on implantation of a PIOL having power PIOLP. The calculation concept is based on either a thick or thin lens model for the cornea and the PIOL. In a Monte-Carlo simulation considering, all PIOL steps listed in the US patent 5,913,898, nonlinear regression models for REF and Δ*M* were defined for each PIOL datapoint.

**Results:**

The calculation shows that simplifying the PIOL to a thin lens could cause some inaccuracies in REF (up to ½ dpt) and Δ*M* for PIOLs with high positive power. The full range of listed ICL powers (− 17 to 17 dpt) could correct REF in a range from − 17 to 12 dpt with a change in Δ*M* from 17 to − 25%. The linear regression considering anterior segment biometric data and the PIOLP was not capable of properly characterizing REF and Δ*M*, whereas the nonlinear model with a quadratic term for the PIOLP showed a good performance for both REF and Δ*M* prediction.

**Conclusion:**

Where PIOL design data are available, the calculation concept should consider the PIOL as thick lens model. For daily use, a nonlinear regression model can properly predict REF and Δ*M* for the entire range of PIOL steps if a vergence calculation is unavailable.


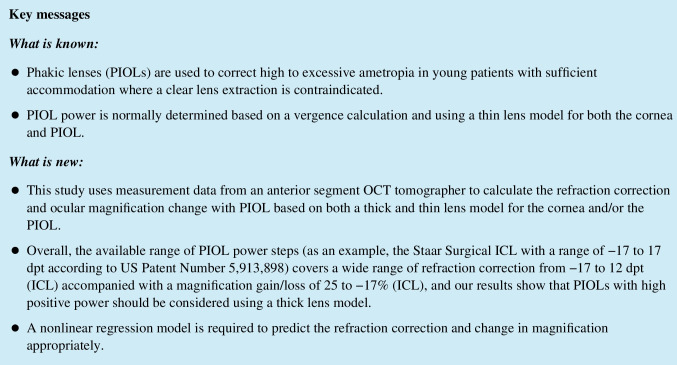


## Introduction

Phakic or pseudophakic supplementary lenses are used to adjust the refraction of the eye in situations where a lens extraction or corneal refractive surgery is not indicated or as an alternative option to corneal refractive surgery. Phakic lenses (PIOL) are mostly implanted in the eyes of young patients with high ametropia (myopia or hyperopia) where laser refractive surgery is not indicated (e.g., due to a thin cornea or high or excessive ametropia) and a clear lens extraction is not proposed as the eye still shows some phakic accommodation [1–5]. In contrast, pseudophakic lenses (addOn) are used after cataract surgery involving implantation of a capsular bag lens for finetuning of the refraction or maintaining pseudophakic multifocality (bifocal or multifocal addOn) or a correction of astigmatism (toric addOn). Therefore, the refractive correction of PIOLs is typically large, whereas the correction of addOn lenses is typically reserved for low power corrections [4].

As a prerequisite, there must be a sufficient space in the posterior chamber of the eye for implantation of the supplementary lens [6–8]. Therefore, IOL manufacturers recommend PIOLs only in situations where the aqueous depth (AQD) measured from the corneal endothelium to the front apex of the crystalline lens is at least 2.8 mm [9]. For pseudophakic eyes, there are typically no space limitations as the capsular bag lens is much thinner than the crystalline lens, and the posterior chamber of the eye gives enough space for implantation of an addOn [4].

PIOL and addOn lenses both have a meniscus lens design with a convex front and a concave back surface. To avoid direct contact between the PIOL and the crystalline lens with a risk of lens opacification or nutrition deficiency of the crystalline lens [10], the periphery of the lens (haptic part) has a special shape which maintains the distance between the PIOL back surface and the crystalline lens to a vault of around 0.4 mm [9]. For addOn, direct contact with the capsular bag lens with a consequence of optical phenomena such as Newton’s rings is also avoided by special haptic configuration [11]. Therefore, the axial position of the supplementary lens (SLPOS; the back surface of the PIOL or addOn with respect to the corneal front apex) is predicted from the ACD as measured with an optical biometer or a tomographer prior to implantation [12]. After implantation, the positioning of the supplementary lens in terms of axial and lateral position as well as tilt can be directly validated using an anterior segment tomographer [13].

Fechner and van der Heijde were the first to present a calculation scheme for supplementary lenses [14–16]. In contrast to replacement lenses where the lens power calculation is based on the biometry of the eye, supplementary lenses are used to shift the pre-existing refraction fully or in part from the spectacle plane to the proposed SLPOS. Therefore, the refraction at the spectacle plane together with the biometry of the anterior eye segment is mandatory [6]. This includes the cornea at least with its front surface radius of curvature (RC_*a*_), but even better with both front and back (RC_*p*_) surface radii and the central corneal thickness (CCT), together with the measurement of the ACD or AQD [17, 18]. Since PIOL implantation typically relates to a large amount of ametropia, we should also record the back vertex distance of the spectacle correction during refractometry to customize the refraction transfer from the spectacle plane to the corneal plane [19]. Current calculation concepts in their simplest form work either with a look-up table for translating spectacle refraction (REF) to the respective power of the supplementary lens or using a vergence calculation based on a thin lens model where the supplementary lens is considered to be located at the predicted SLPOS [9].

The purpose of this study was the following

- to develop a vergence-based calculation concept to determine the power of PIOL, the refraction correction at the spectacle plane, and the change in lateral magnification using anterior segment OCT data and a thick and thin lens model for the cornea and the PIOL,
- to apply this calculation concept to a large clinical dataset with anterior segment OCT measurements performed with the Casia2 and using the “real” design data of a PIOL as disclosed in US patent 5,913,898 (ICL, Staar Surgical), and
- to derive Monte-Carlo prediction models for refractive correction and lateral magnification using real ICL design data and the Casia2 anterior segment measurement data.

## Materials and methods

### Dataset for analysis

In this retrospective study, we analyzed a dataset containing measurements from 5224 untreated eyes from the Augen- und Laserklinik Castrop-Rauxel, Castrop-Rauxel, Germany which was transferred to us. The local ethics committee (Ärztekammer des Saarlandes) provided a waiver for this study (157/21). The dataset contains patient ID, age, sex, eye side, and anterior segment OCT measurements performed with the Casia2 (Tomey GmbH, Nürnberg, Germany, software version Ver.50.5A.03). The raw data (.XLSX-format) were transferred to us in an anonymized fashion (with a randomly generated patient ID), precluding back-tracing of the patient. The CSV data were imported into MATLAB (Matlab 2021a, MathWorks, Natick, USA) for further processing.

### Preprocessing of the data and data selection

Custom software was written in Matlab. The dataset included radius of curvature of the corneal front surface (RC1_*a*_/RC2_*a*_ in mm in the flat/steep corneal meridian with axis A1_*a*_/A2_*a*_ in °) and back surface (RC1_*p*_/RC2_*p*_ in mm in the flat/steep corneal meridian with axis A1_*p*_/A2_*p*_ in °), central corneal thickness (CCT in mm), aqueous depth (AQD in mm), anterior chamber depth (ACD = AQD + CCT in mm), and the central thickness of the crystalline lens (LT in mm). The mean corneal radius of curvature was calculated from the CASIA data for the front surface (RC_*a*_ = ½ (RC1_*a*_ + RC2_*a*_)) and back surface (RC_*p*_ = ½ (RC1_*p*_ + RC2_*p*_)). For the refractive index of cornea, we used *n*_*C*_ = 1.376 as specified in the schematic model eye of Liou & Brennan [20]. For considering the cornea as thin lens, a keratometer index of *n*_*K*_ = 1.3375 was used to convert the corneal front surface radius into corneal power PC (PC = (*n*_*K*_-1)/RC_*a*_).

Where measurements of both eyes of one individual were available in the dataset, one eye was selected based on a random code and the second eye was discarded. Repeat measurements of the same eye were also discarded from the dataset. Finally, only eyes with an AQD of at least 2.8 mm were considered for this study according to the recommendations of the manufacturer of the PIOL.

### PIOL design data

For modelling, we used the design data of the Staar Surgical ICL since—to our knowledge—this is the only disclosed lens design for PIOLs. The design data for the ICL were taken from United States Patent IP 5,913,898 (published 22 Jun, 1999). In this patent, for lenses with a positive equivalent power (PIOLP), the central PIOL thickness (PIOLT) and the PIOL front surface radius (PIOLR_*a*_) are provided for power steps from PIOLP = 3.0 (0.5) 17.0 dpt (Fig. 7 on sheet 3). For lenses with a negative equivalent power PIOLP, the central PIOL thickness PIOLT and the PIOL front surface radius (PIOLR_*a*_) and back surface radius (PIOLR_*p*_) are provided for power steps from PIOLP =  − 3.0 (− 0.5) − 17.0 dpt (Fig. 14 on sheet 6). From the design data of the PIOL with negative PIOLP, the refractive index (*n*_PIOL_) was back-calculated based on the Gullstrand formula assuming a refractive index of aqueous humor of *n*_*A*_ = 1.336 derived from the schematic model eye of Liou & Brennan [20]. From the 29 PIOL designs with negative PIOLP, we obtain a refractive index of *n*_PIOL_ = 1.4490 ± 0.0000; median 1.4490; 95% confidence interval with a lower limit of 1.4489, and an upper limit of 1.4491. The mean refractive index *n*_PIOL_ = 1.4490 was used to back-calculate the missing data for the PIOL back surface radius RPIOL_*p*_ for the 29 PIOL designs with positive PIOLP (as these are not listed in the patent).

### Calculation of PIOL power, refraction correction, and change in lateral magnification

For all measurements in the dataset, the refraction correction for the entire range of PIOL power values (using the example of the ICL, in total 58 power steps; PIOL with PIOLP =  − 17.0 (0.5) − 3.0 dpt and PIOLP = 3.0 (0.5) 17.0 dpt) was considered in this Monte-Carlo simulation. We considered four scenarios, with different combinations of thick and thin lens models for the cornea and PIOL:

- A: thick lens cornea with front/back surface radius RC_*a*_/RC_*p*_ and a central corneal thickness CCT (refractive index *n*_*C*_) together with a thick lens PIOL (front and back surface curvature and central thickness as specified in the patent with missing data back-calculated);
- B: thin lens cornea with front surface radius RC_*a*_ converted to corneal power using *n*_*K*_ together with a thick lens PIOL (front and back surface curvature and central thickness as specified in the patent with missing data back-calculated);
- C: thick lens cornea with front/back surface radius RC_*a*_/RC_*p*_ and a central corneal thickness CCT (refractive index *n*_*C*_) together with a thin lens PIOL specified with the nominal equivalent power PIOLP; and
- D: thin lens cornea with front surface radius RC_*a*_ converted to corneal power using *n*_*K*_ together with a thin lens PIOL specified with the nominal equivalent power PIOLP.
- For situations A and B/C and D, we assumed that the PIOL back surface/thin lens PIOL was located at a distance SLPOS = (ACD – vault) behind the corneal front vertex (or SLPOS = (AQD – vault) behind the corneal back vertex). VD was assumed to be 12 mm for all calculations.

**Fig. 1:**
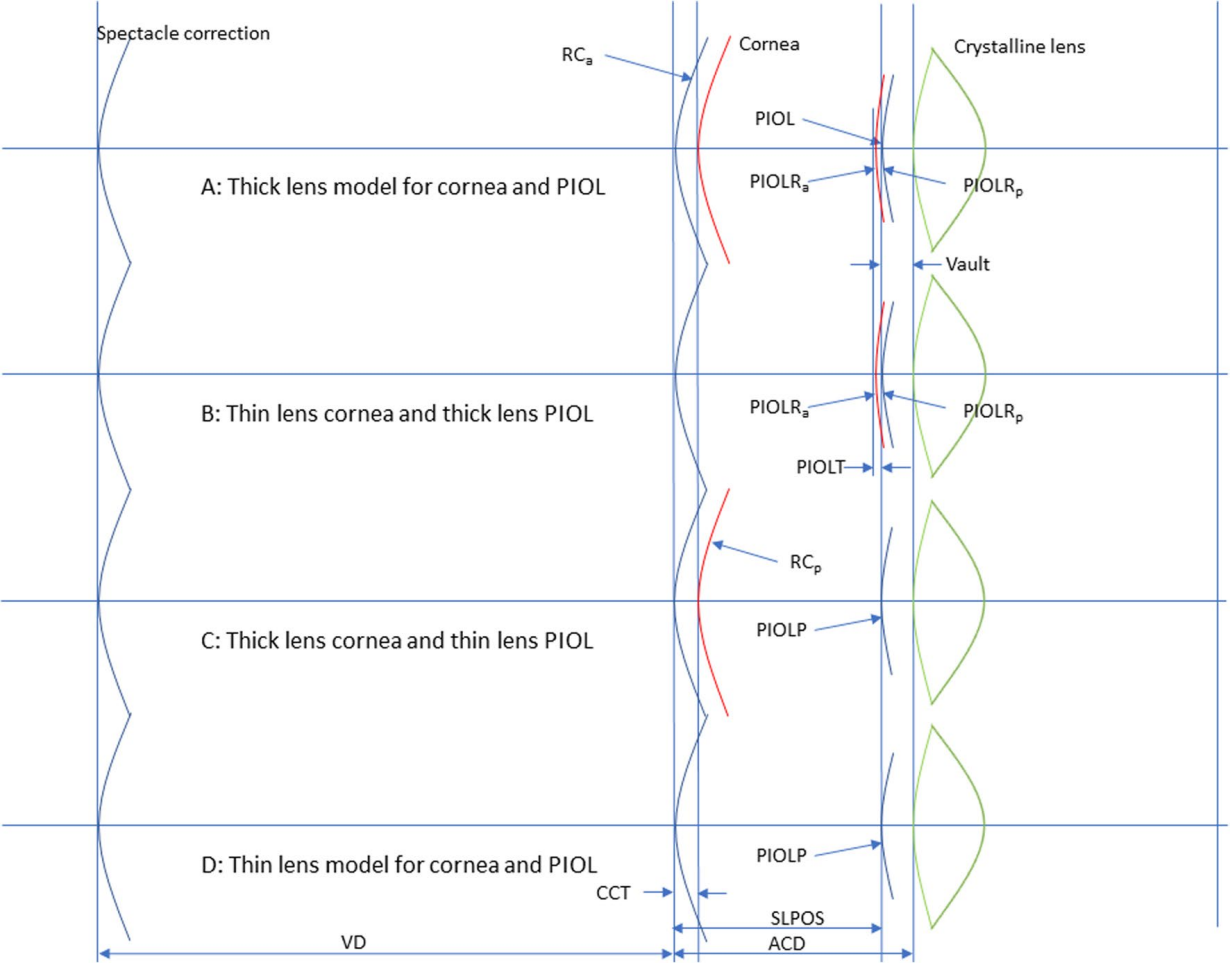
Schematic drawing of the phakic model eye used for calculation of phakic lens power or refraction correction after implantation of a PIOL. Scenario (**A**) refers to a thick lens model for both the cornea and the PIOL, scenario (**B**) to a thin lens cornea and a thick lens PIOL, scenario (**C**) to a thick lens cornea and a thin lens PIOL, and scenario (**D**) to a thin lens model for both the cornea and the PIOL. RC_*a*_/RC_*p*_ refers to the corneal front/back surface radius, CCT to the central corneal thickness, ACD/AQD to the anterior chamber/aqueous depth, SLPOS to the axial position of the PIOL back surface plane (thick PIOL) or the position of the thin PIOL, vault to the distance between the PIOL and the crystalline lens front vertex, PIOLP to the power of the PIOL, and PIOLR_*a*_/PIOLR_*p*_ to the curvature of the PIOL front/back surface

Figure 1 displays the optical scheme of the model eye used for PIOL power calculation, calculation of the refractive correction with the PIOL, and lateral magnification change as the ratio of PIOL corrected to spectacle corrected eye. For calculation of refraction correction of the PIOL at the spectacle plane REF, we assume that the eye is fully corrected in the postoperative situation (with the PIOL). For this condition, we calculate the vergence at SLPOS plane (*V*_SLPOS_). This vergence is traced back for the preoperative situation (without PIOL) to the spectacle plane to read out the refraction correction of the PIOL at the spectacle plane REF.

Explicitly, the refractive correction REF at the spectacle plane if a thick or thin lens PIOL with equivalent power PIOLP is implanted is given by the following:

Scenario A:


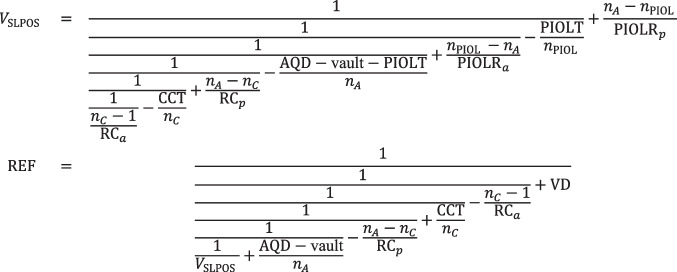


Scenario B: 
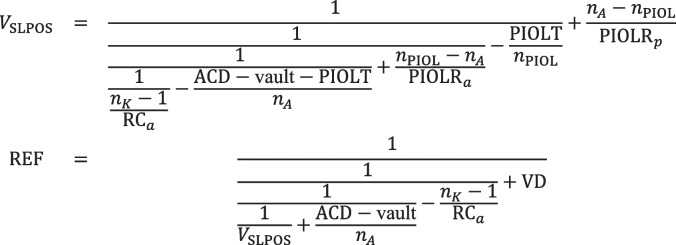


Scenario C: 
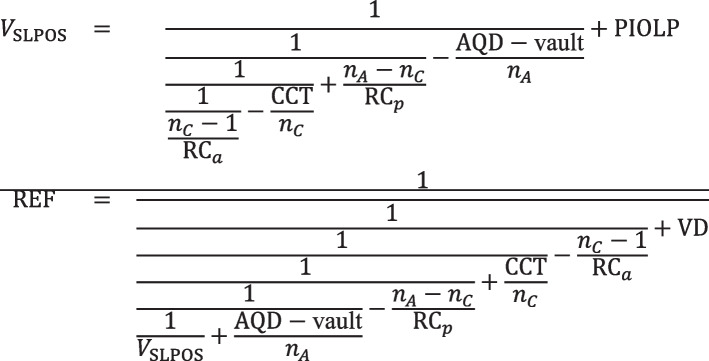


Scenario D: 
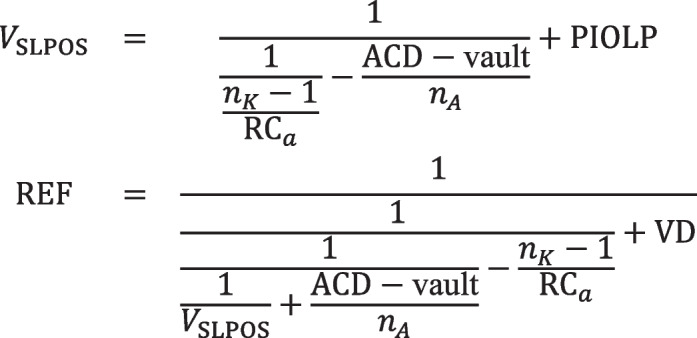


The relative change in lateral magnification (Δ*M* in %) when the spectacle correction (preoperatively) is replaced by the refractive correction with the PIOL (postoperatively) can be easily derived from the product of all vergences in front of (Π*V*_*pr*_) and behind the refractive surfaces (Π*V*′_*pr*_) from the preoperative situation as well as the product of all vergences in front of (Π*V*_*po*_) and behind the refractive surfaces (Π*V*′_*po*_) from the postoperative situation as shown in Langenbucher et al. [23]. Explicitly, the change in lateral magnification Δ*M* resulting from implantation of a PIOL replacing the preoperative spectacle correction for scenarios A to D reads:

$$\Delta M=100\bullet \left(\frac{\prod {V}_{po}}{\prod {V{\prime}}_{po}}\bullet \frac{\prod {V{\prime}}_{pr}}{\prod {V}_{pr}}-1\right)$$

The applicability of this calculation concept is shown in a clinical example. Using either the corneal front surface radius of curvature data (keratometry, cornea considered as a thin lens) or corneal front and back surface curvature data together with the corneal thickness (cornea considered as a thick lens), the refraction correction REF for all 58 power steps of the PIOL (in the case of the Staar Surgical ICL), considered either as a thin lens with (labelled) equivalent power PIOLP or as a thick lens using the design data derived from the patent) is calculated, and the change in ocular magnification Δ*M* resulting from replacement of the spectacle correction REF with a PIOL can be predicted.

### Monte-Carlo prediction model

In the next step, values of REF and Δ*M* are calculated from all clinical measurements in the dataset for all PIOL power steps. Using these data, prediction models are defined for scenarios A and B (thick lens or thin lens cornea and a thick lens PIOL defined by the design data) to determine REF and Δ*M* from the biometric measures. The relevant predictors for the models are identified using a stepwise regression method which adds or removes potential predictors according to their performance in the model. As potential predictors, we considered RC_*a*_, RC_*p*_, and CCT (for scenario A), PC (for scenario B), ACD, LT, and PIOLP. The stepwise regression algorithm [21, 22] was initialized with a constant model and restricted to first- and second-order terms (linear and quadratic quantities) without interactions. Depending on the significance level (*p* value), terms were added (if *p* ≤ 0.01) or removed (if *p* ≥ 0.1) from the model. The final model was defined by the regression coefficients and the respective *p* values. To evaluate the model performance of the final models, we recorded the root-mean-squared prediction error, the adjusted coefficient of determination *R*^2^, and the *F* statistics (F and *p* value) comparing the final model to the initial constant model [21].

## Results

The dataset transferred to us contained *N* = 5224 measurements made using a Casia 2 anterior segment tomographer. After random selection of one eye per individual, discarding duplicate measurements of one eye, and filtering for AQD values (AQD ≥ 2.8 mm), *N* = 2365 data points were considered for our Monte Carlo simulation. (1537 right eyes and 828 left eyes from 1264 female and 1101 male patients). The descriptive statistics including mean, standard deviation (SD), median, and 95% confidence interval (with the 2.5% and 97.5 quantile as lower and upper boundaries) of the relevant input parameters are listed in Table 1.

**Table 1:** Descriptive data of the relevant parameters derived from the Casia 2 anterior segment tomographer. RC_*a*_ and RC_*p*_ refer to the corneal front and back surface radii of curvature, CCT to the central corneal thickness, PC to the keratometric power converted from RC_*a*_ using a keratometer index of *n*_*K*_ = 1.3375, AQD to the aqueous depth measured from the corneal endothelium to the crystalline lens front vertex, ACD to the anterior chamber depth measured from the corneal to the crystalline lens front apex, and LT to the central thickness of the crystalline lens. Data are given in terms of the mean, standard deviation (SD), median, and the lower (2.5% quantile) and upper (97.5% quantile) boundaries of the 95% confidence interval

*N* = 2365	RC_*a*_ in mm	RC_*p*_ in mm	CCT in mm	PC in dpt	AQD in mm	ACD in mm	LT in mm
Mean	7.7633	6.5751	0.5434	43.0693	3.1256	3.669	3.9217
SD	0.2854	0.2473	0.0366	1.5421	0.2381	0.2362	0.2024
Median	7.7300	6.5700	0.5440	43.1586	3.0870	3.6270	3.9221
2.5% quantile	7.2700	6.1000	0.4670	40.0178	2.8130	3.3520	3.5349
97.5% quantile	8.3500	7.0637	0.6130	45.9809	3.6894	4.2351	4.3289

**Fig. 2:**
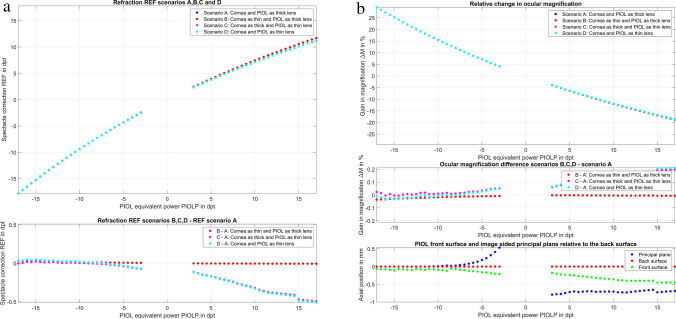
**a** Clinical example calculation of the refractive correction (REF) at the spectacle plane vs. equivalent PIOL power (PIOLP) with four different models (scenarios A to D, upper plot) and the difference in REF between the simplified models (scenarios B, C, D) compared to the thick lens model of cornea and PIOL (scenario A, lower plot). **b** Change in lateral magnification Δ*M* with an PIOL implantation is shown for scenarios A to D (upper plot), the differences in Δ*M* between the scenarios B, C, D and scenario A (middle plot), and the axial position of the front surface and the image-sided principal plane of the ICL with respect to the back surface (lower plot) is shown for PIOL power steps PIOLP = − 17.0 (0.5) − 3.0 dpt and 3.0 (0.5) 17.0 dpt

In the clinical example, we used the corneal data derived from the Liou & Brennan schematic model eye [20] with RC_*a*_ = 7.77 mm, RC_*p*_ = 6.40 mm, and CCT = 0.50 mm for the thick lens cornea or PC = 337.5/7.77 = 43.44 dpt. The AQD, vault, and back vertex distance of the spectacle correction were assumed to be 3.00 mm, 0.40 mm, and 14.00 mm respectively. For all power steps of the ICL shown in the patent (PIOLP =  − 17.0 (0.5) − 3.0 and 3.0 (0.5) 17.0 dpt), the respective refraction correction at the spectacle plane REF was calculated for scenarios A, B, C, and D as shown in Fig. 2a in the upper graph. The lower graph shows the differences in REF considering the simplifications in the model made in scenarios B, C, and D as compared to scenario A (with both cornea and PIOL treated as a thick lens). From the graph, we can see that for PIOLs with negative power PICL, there is only a very slight difference between scenarios C and A and D and A (both thin lens models for the ICL) and no significant difference between B and A (both thick lens models for the PIOL). For PIOLs with positive power PPIOLP, the models with a thin lens PIOL (scenarios C and D) deviate further and further from those with a thick lens PIOL (scenarios A and B) with increasing PIOLP as a result of increasing PIOL thickness PIOLT. In all cases, the differences between B and A are small to negligible. The upper graph in Fig. 2b shows the change in relative magnification Δ*M* for scenarios A, B, C, and D, and the middle graph shows the differences of Δ*M* comparing Δ*M* based on the model simplifications made in scenarios B, C, and D compared to scenario A with a thick lens cornea and PIOL. From the graphs, we can see that there is no clinically relevant difference in Δ*M* comparing the four scenarios. For myopic corrections with a PIOL. there is a systematic increase in magnification with absolute lens power, whereas for hyperopic corrections with a PIOL, there is a corresponding reduction in magnification as lens power increases. In the lower graph of Fig. 2b, the PIOL front surface position (green dots) and the image side principal plane positions for the PIOL (blue dots, both referenced to the position of the PIOL back surface plane SLPOS) are plotted for all PIOL power steps based on the design data given in the patent. For PIOLs with a negative power PIOLP, the principal plane is strictly behind the PIOL back surface, and for PIOLs with a positive power PIOLP, the principal plane is strictly located in front of the PIOL front surface. For lower power values of the PIOL, the PIOL principal plane is more distant from the respective PIOL surface.

Having noted differences in the REF characteristics between the thick lens model (scenarios A and B) and the thin lens model (scenarios C and D) for the ICL (as shown in Fig. 2), we subsequently restricted our models for prediction of REF and Δ*M* in our Monte-Carlo simulation to scenarios A and B (thick and thin lens cornea and thick lens PIOL).

### Regression models for prediction of refraction correction REF

Scenario A (thick lens cornea and PIOL):

For scenario A, the linear regression model for prediction of REF includes an intercept (*p* = 6.76·10^−21^) and also the predictors RC_*a*_ (*p* = 2.50·10^−27^), ACD (*p* = 2.49·10^−19^), and PIOLP (*p* < 1.00·10^−200^). The model definition reads as follows:

$${\text{REF}}=-0.63189-0.082719\bullet {{\text{RC}}}_{a} \left[{\text{mm}}\right]+0.082956\bullet {\text{ACD}} \left[{\text{mm}}\right]+0.83597\bullet {\text{PIOLP}} \left[{\text{dpt}}\right]$$

The model performance is given by a root-mean-squared prediction error of 0.807 dpt, a coefficient of determination of *R*^2^ = 0.992, and an *F* statistic (comparing the final model to a constant model) of *F* = 5.77·10^6^/*p* < 10^−200^.

Accepting linear and quadratic terms in the regression, the nonlinear regression model for prediction of REF includes an intercept (*p* = 1.82·10^−163^) and the predictors RC_*a*_ (*p* < 1.00·10^−200^), ACD (*p* = 7.99·10^−193^), PIOLP (*p* < 1.00·10^−200^), and PIOLP^2^ (*p* < 1.00·10^−200^). The model definition reads as follows:

$${\text{REF}}=0.44816-0.082719\bullet {{\text{RC}}}_{a} \left[{\text{mm}}\right]+0.082956\bullet {\text{ACD}} \left[{\text{mm}}\right]+0.83597\bullet {\text{PIOLP}} \left[{\text{dpt}}\right]-0.0091919\bullet {{\text{PIOLP}}}^{2} \left[{\text{dpt}}^{2}\right]$$

The model performance is given by a root-mean-squared prediction error of 0.196 dpt, a coefficient of determination of *R*^2^ = 1.000, and an *F* statistic (comparing the final model to a constant model) of *F* = 7.35·10^7^/*p* < 10^−200^.

Scenario B (thin lens cornea and thick lens PIOL):

For scenario B, the linear regression model for prediction of REF includes an intercept (*p* = 1.16·10^−164^) and the predictors PC (*p* = 2.79·10^−26^), ACD (*p* = 8.99·10^−21^), and PIOLP (*p* < 1.00·10^−200^). The model definition reads as follows:

$${\text{REF}}=-1.9212+0.014728\bullet {\text{PC}} \left[{\text{dpt}}\right]+0.08603\bullet {\text{ACD}} \left[{\text{mm}}\right]+0.83512\bullet {\text{PIOLP}} \left[{\text{dpt}}\right]$$

The model performance is given by a root-mean-squared prediction error of 0.805 dpt, a coefficient of determination of *R*^2^ = 0.992, and an *F* statistic (comparing the final model to a constant model) of *F* = 5.79·10^6^/*p* < 10^−200^.

Accepting linear and quadratic terms in the regression, the nonlinear regression model for prediction of REF includes an intercept (*p* = 10^−200^) and the predictors PC (*p* < 10^−200^), ACD (*p* < 10^−200^), PIOLP (*p* < 10^−200^), and PIOLP^2^ (*p* < 1.00·10^−200^). The model definition reads as follows:

$${\text{REF}}=-0.84481+0.014728\bullet {\text{PC}} \left[{\text{dpt}}\right]+0.08603\bullet {\text{ACD}} \left[{\text{mm}}\right]+0.83512\bullet {\text{PIOLP}} \left[{\text{dpt}}\right]-0.0091611\bullet {{\text{PIOLP}}}^{2} \left[{\text{dpt}}^{2}\right]$$

The model performance is given by a root-mean-squared prediction error of 0.198 dpt, a coefficient of determination of *R*^2^ = 0.999, and an *F* statistic (comparing the final model to a constant model) of *F* = 7.21·10^7^/*p* < 10^−200^.

**Fig. 3:**
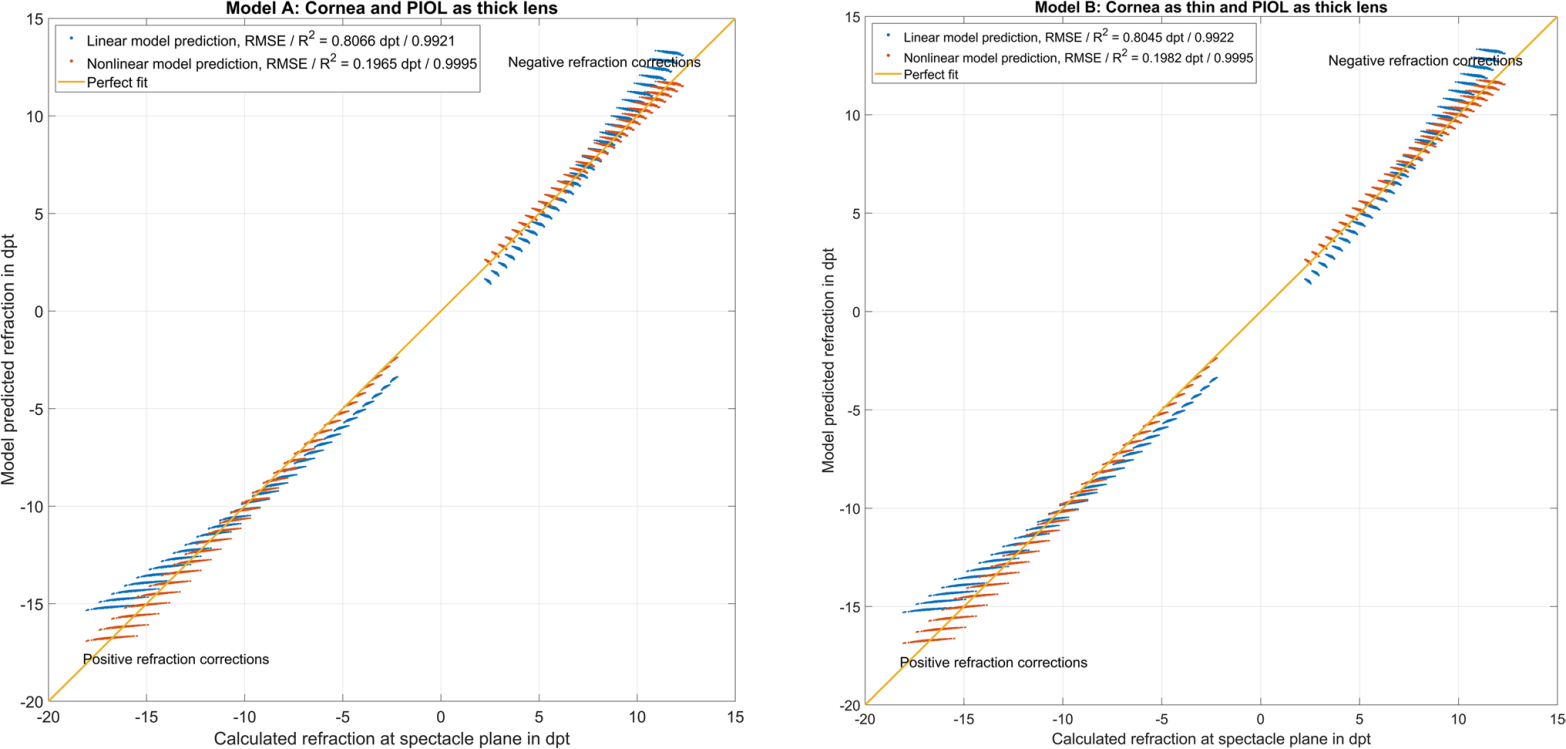
Performance of the linear (in blue) and nonlinear (quadratic, in red) prediction models to estimate the refractive correction REF at the spectacle plane with implantation of a phakic intraocular lens (PIOL), together with the best fit line. The left plot refers to scenario A with a thick lens model for both the cornea and the PIOL and the right graph to a simplification to a thin lens cornea (with keratometric power PC) and a thick lens PIOL. The linear model appears to be an oversimplification, but the quadratic model shows a good overall performance. The root-mean-squared prediction error and the coefficient of determination are mentioned in the legend

Figure 3 shows the performance of the regression model based REF vs. REF derived from a vergence calculation for scenarios A and B. In the upper/lower graph referring to scenario A/B, the performance of the linear and the nonlinear (quadratic) regression model are displayed. We can see directly from the graph that the linear model (in blue) shows some oversimplification with an underestimation of the absolute REF for large negative and small positive refractive corrections and an overestimation of absolute REF for large positive and small negative refractive corrections. In contrast, the nonlinear model (in red) shows an overall good performance and could be directly used for clinical purposes.

### Regression models for prediction of change in relative magnification ΔM

Scenario A (thick lens cornea and PIOL):

For scenario A, the linear regression model for prediction of Δ*M* includes an intercept (*p* = 6.11·10^−13^) and the predictors RC_*a*_ (*p* = 1.21·10^−26^), and PIOLP (*p* < 1.00·10^−200^). The model definition reads as follows:

$$\Delta M=0.65142+0.12448\bullet {{\text{RC}}}_{a} \left[{\text{mm}}\right]-1.2113\bullet {\text{PIOLP}} \left[{\text{dpt}}\right]$$

The model performance is given by a root-mean-squared prediction error of 1.23%, a coefficient of determination of *R*^2^ = 0.991, and an *F* statistic (comparing the final model to a constant model) of *F* = 7.80·10^6^/*p* < 10^−200^.

Accepting linear and quadratic terms in the regression, the nonlinear regression model for prediction of Δ*M* includes an intercept (*p* < 10^−200^) and the predictors RC_*a*_ (*p* < 10^−200^), RC_*p*_ (*p* = 6.76·10^−5^), ACD (*p* = 1.68·10^−62^), PIOLP (*p* < 1.00·10^−200^), and PIOLP^2^ (*p* < 1.00·10^−200^). The model definition reads as follows:

$$\Delta M=-0.89457+0.13309\bullet {{\text{RC}}}_{a} \left[{\text{mm}}\right]-0.011804\bullet {{\text{RC}}}_{p} \left[{\text{mm}}\right]-0.033995\bullet {\text{ACD}} \left[{\text{mm}}\right]-1.2113\bullet {\text{PIOLP}} \left[{\text{dpt}}\right]+0.014311\bullet {{\text{PIOLP}}}^{2} \left[{\text{dpt}}^{2}\right]$$

The model performance is given by a root-mean-squared prediction error of 0.178%, a coefficient of determination of *R*^2^ = 1.000, and an *F* statistic (comparing the final model to a constant model) of *F* = 1.51·10^8^/*p* < 10^−200^.

Scenario B (thin lens cornea and thick lens PIOL):

For scenario B, the linear regression model for prediction of Δ*M* includes an intercept (*p* = 6.86·10^−143^) and the predictors PC (*p* = 4.61·10^−26^), ACD (*p* = 5.11·10^−3^), and PIOLP (*p* < 1.00·10^−200^). The model definition reads as follows:

$$\Delta M=2.7297-0.022375\bullet {\text{PC}} \left[{\text{dpt}}\right]-0.039317\bullet {\text{ACD}} \left[{\text{mm}}\right]-1.2109\bullet {\text{PIOLP}} \left[{\text{dpt}}\right]$$

The model performance is given by a root-mean-squared prediction error of 1.23%, a coefficient of determination of *R*^2^ = 0.991, and an *F* statistic (comparing the final model to a constant model) of *F* = 5.23·10^6^ / *p* < 10^−200^.

Accepting linear and quadratic terms in the regression, the nonlinear regression model for prediction of Δ*M* includes an intercept (*p* = 10^−200^) and the predictors PC (*p* < 10^−200^), ACD (*p* = 4.74 10^−84^), PIOLP (p < 10^−200^), and PIOLP^2^ (*p* < 1.00·10^−200^). The model definition reads as follows:

$$\Delta M=1.0508-0.022375\bullet \mathrm{PC }\left[{\text{dpt}}\right]-0.0393176\bullet {\text{ACD}} \left[{\text{mm}}\right]-1.2109\bullet {\text{PIOLP}} \left[{\text{dpt}}\right]+0.014273\bullet {{\text{PIOLP}}}^{2} \left[{\text{dpt}}^{2}\right]$$

The model performance is given with a root-mean-squared prediction error of 0.177%, a coefficient of determination of *R*^2^ = 1.000, and an *F* statistic (comparing the final model to a constant model) of *F* = 1.91·10^8^/*p* < 10^−200^.

**Fig. 4:**
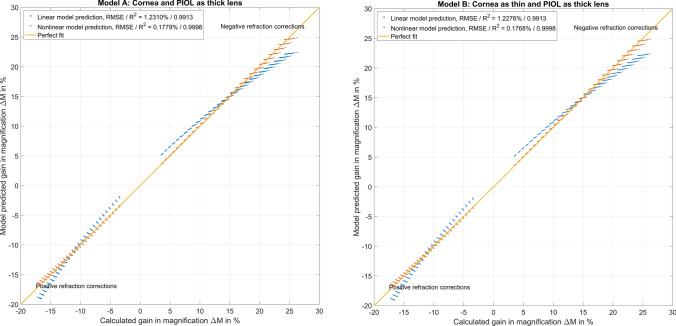
Performance of the linear (in blue) and nonlinear (quadratic, in red) prediction model to estimate the change in relative lateral magnification of the eye Δ*M* with implantation of a phakic intraocular lens (PIOL), together with the best fit line. The upper plot refers to scenario A with a thick lens model for both the cornea and the PIOL and the lower graph to a simplification to a thin lens cornea (with keratometric power PC) and a thick lens PIOL. The linear model appears to be an oversimplification, but the quadratic model shows a good overall performance. The root-mean-squared prediction error and the coefficient of determination are mentioned in the legend. Please note that negative values of Δ*M* refer to a hyperopic correction

Figure 4 shows the performance of the linear and nonlinear regression model-based Δ*M* vs. Δ*M* derived from a vergence calculation for scenarios A (upper graph) and B (lower graph). We can directly see from the graph that the linear model (in blue) shows some oversimplification with an underestimation of the absolute value of Δ*M* for large negative and small positive refractive corrections and an overestimation of absolute Δ*M* for large positive and small negative refractive corrections. In contrast, the nonlinear model (in red) shows an overall good performance and could be directly used for clinical purposes.

## Discussion

Supplementary lenses are typically implanted in a phakic eye to maintain phakic accommodation or into a pseudophakic eye for adjustment of the patient’s refraction or providing some features such as astigmatic correction or pseudoaccommodation using a multifocal design. Even though the calculation concept is identical for both applications, the correction is quite different: in PIOL implantation, we typically have young patients with sufficient accommodation and a large ametropia which cannot be corrected by laser refractive surgery [1, 3, 5, 12] or as an alternative to laser refractive surgery, whereas in addOn implantation, we focus mostly on finetuning of the refractive correction and probably adding some features for enhanced patient comfort [4].

The standard way of calculating PIOL power is to define the axial position (SLPOS) using ACD or AQD measurement and a prediction of the vault and to consider the PIOL as a thin lens at SLPOS. Then we calculate for the postoperative situation (with or without some residual spectacle refraction) the vergence at the SLPOS plane with the PIOL and trace backwards to the spectacle plane (preoperative situation) without considering the PIOL. From this concept, we derive the change in spectacle refraction if a PIOL with power PIOLP is implanted [19, 23].

In the present paper, we have considered the PIOL using a thick lens model. Our study was based on design data of the ICL as published in a patent publication from 1999. Since the specifications were incomplete, some of the missing data in the listing of the lens design had to be calculated. In the case of the negative powered PIOLs, the front and back surface radii and the central thickness were available, which allowed for a back-calculation of the refractive index (with a very small variation mostly due to the data precision in the listing being restricted to two decimals). Using the same refractive index for the positive powered PIOLs, it was possible to derive the back surface radius for each power step as a function of the published front surface radius and central thickness. With these calculations, all relevant data for the full power range from − 17 to − 3 dpt and from 3 to 17 dpt in ½ dpt steps were available.

In contrast to a thin lens model of the PIOL where PIOLP can be directly calculated from the intended refraction change from pre- to postoperative and the biometric measures of the anterior eye segment, the procedure with a thick lens model is somewhat different: here we calculate the refraction at the spectacle plane REF for each power step of the PIOL and select the most appropriate PIOLP value from all the options.

To investigate the differences between a PIOL calculation using a thick or thin lens model for the cornea or the PIOL, we set up four different models: In scenario A, we defined both the cornea and the PIOL as a thick lens and used corneal tomography data from the cornea and the design data specified in the patent to calculate the refraction correction for each PIOLP value at the spectacle plane. In scenario B, we simplified the cornea to a thin lens specified by its keratometric power (based on a keratometer index *n*_*K*_ = 1.3375) to evaluate the differences to scenario A. In addition, we defined the PIOL as a thin lens model and combined this with a thick lens cornea (scenario C) or a thin lens cornea (scenario D) to investigate potential differences between the models if the corneal back surface curvature and the corneal thickness are not known.

After deriving the vergence representation for all four scenarios, the relative lateral magnification (for objects at infinity) was derived from all vergences in front of and behind the refracting surfaces preoperatively (spectacle correction and cornea) and postoperatively (cornea and PIOL) assuming a full refractive correction with the PIOL [23]. This simple concept of estimating the lateral magnification is typically used for predicting aniseikonia or the surgically induced change in magnification after cataract surgery with capsular bag lens implantation or after laser refractive surgery.

Typically, the central thickness of the PIOL is very small, especially in minus powered PIOLs. Therefore, we would not expect too much difference between a thick lens and thin lens model of the PIOL. However, as our data show, for positive powered PIOLs, the central PIOL thickness PIOLT cannot be fully neglected in our calculation concept. As we are dealing strictly with meniscus lenses having a convex front and concave back surface, the image side principal plane of the PIOL is in front of the PIOL front vertex for positive powered PIOLs and behind the back vertex for negative powered PIOLs as shown in Fig. 2 in the lower graph. For low powered PIOLs (positive or negative), the distance of the principal plane is more distant from the lens surfaces. As the nominal PIOL power labelled on the PIOL refers to the equivalent power according to ISO standards (ISO 11979: 2018), the thin lens PIOL model should no longer be considered at the SLPOS plane. Luckily, this displacement effect due to the position of the image side principal plane is rather small for low powered PIOLs, but it does add some nonlinearity to the translation of PIOL to REF as well as Δ*M*, making a representation with a linear prediction model inaccurate.

From the clinical example shown in Fig. 2, we see that there is no clinically relevant difference in REF as derived with either a thick or thin lens model of the cornea, but that there is some difference between the thick and thin lens models of the PIOL, especially with larger positive PIOLP (due to a larger central PIOL thickness PIOLT). Therefore, as the PIOL design data were available, we decided to use for our Monte-Carlo prediction model for REF and Δ*M* the thick lens PIOL in combination with a thick lens model for the cornea (scenario A, where tomography data are available) or a simplified model of the cornea as thin lens (scenario B, where only keratometric power PC from corneal front surface measurement is available).

Using a stepwise regression method [21, 22], we identified the relevant predictors for the model. When restricted to a linear model without interactions, we found that the model prediction was not sufficient. We therefore generalized our prediction models by allowing first-order and second-order terms without interactions (linear and quadratic terms). The root-mean-squared prediction error of all nonlinear models for REF and Δ*M* for scenarios A and B was systematically lower compared to the respective linear models, as can be clearly seen from the graphs in Figs. 3 and 4. This means that clinicians could use either the vergence calculation as shown in the Methods section or the nonlinear regression models considering linear and quadratic terms for PIOLP, since both calculations yield a sufficient precision for clinical routine application.

As a consequence of the present study, we see that simplifying the PIOL calculation to a thin lens PIOL model causes some error, especially for PIOLs with larger positive power due to the larger PIOLT values. This underestimation of the refractive correction at the spectacle plane is mostly in a range of around ½ dpt. For negative powered PIOLs, the refractive correction at the spectacle plane is slightly lower/higher than PIOLP for low/high PIOLP, and for positive powered PIOLs, the refractive correction at the spectacle plane is systematically less than the PIOLP. PIOLs with negative power cause a gain of magnification up to 25% (for PIOLP =  − 17 dpt), whereas PIOLs with positive power cause a loss of magnification of up 17% (for PIOLP = 17 dpt).

Our study shows some limitations: The listing of ICL design data from the specific patent considered here does not necessarily represent the shape of all PIOL models currently on the market. The ICL design and the delivery range have been changed over time, and other manufacturers of PIOLs might use different designs. Consequently, the refraction correction and the magnification change might differ slightly depending on the PIOL design. In our calculation, we had to infer some values: the refractive index of the PIOL material *n*_PIOL_ was back-calculated from the design data of the negative power PIOLs, and the back surface curvature of the positive power PIOLs was then back-calculated from the equivalent power and the calculated refractive index. If a full set of lens design data including the refractive index were available, the calculation scheme could directly use these data for calculation of REF and Δ*M* [17, 18, 24]. We used a vergence calculation which is restricted to the paraxial Gaussian space. For large ray angles, the concept might show some inaccuracies [25, 26]. And last but not least, we assumed that the PIOL will be positioned at a fixed distance (vault) in front of the crystalline lens. A more detailed prediction concept based on biometric measures of the anterior eye segment could further improve the calculation.

In conclusion, in this paper, we presented a concept for calculating the refraction correction for phakic lenses (PIOLs) based on a model eye having a thick or thin lens cornea and a thick or thin lens PIOL. The calculation concept is based on a vergence transformation and could be applied to any PIOL design. In addition to the change in refraction at the spectacle plane, we directly found the gain or loss in ocular magnification resulting from a transfer of the spectacle correction to the PIOL plane. Both the refractive correction at the spectacle plane and the change in magnification could be properly predicted either using direct vergence calculation or with nonlinear regression models (with a quadratic term for the PIOL power), whereas a linear regression model should not be used as it shows some systematic prediction error.
